# Enhanced prediction of renal function decline by replacing waist circumference with “A Body Shape Index (ABSI)” in diagnosing metabolic syndrome: a retrospective cohort study in Japan

**DOI:** 10.1038/s41366-021-01026-7

**Published:** 2021-11-25

**Authors:** Daiji Nagayama, Kentaro Fujishiro, Shinichi Tsuda, Yasuhiro Watanabe, Takashi Yamaguchi, Kenji Suzuki, Atsuhito Saiki, Kohji Shirai

**Affiliations:** 1 Department of Internal Medicine, Nagayama Clinic, 2-12-22, Tenjin-cho, Oyama-city, Tochigi 3230032 Japan; 2 Center of Diabetes, Endocrinology and Metabolism, Toho University, Sakura Medical Center, 564-1, Shimoshizu, Sakura-city, Chiba 2850841 Japan; 3 Japan Health Promotion Foundation, 1-24-4, Ebisu, Shibuya-ku, Tokyo 1500013 Japan; 4 Fukuda Denshi Co., Ltd., 3-39-4, Hongo, Bunkyo-ku Tokyo, 1130033 Japan; 5 Department of Internal Medicine, Mihama Hospital, 1-1-5, Uchise, Mihama-ku Chiba, 2610013 Japan

**Keywords:** Risk factors, Kidney diseases, Body mass index, Epidemiology

## Abstract

**Background:**

Abdominal obesity as a risk factor for diagnosing metabolic syndrome (MetS) is conventionally evaluated using waist circumference (WC), although WC does not necessarily reflect visceral adiposity.

**Objective:**

To examine whether replacing WC with “A Body Shape Index (ABSI)”, an abdominal obesity index calculated by dividing WC by an allometric regression of weight and height, in MetS diagnosis is useful for predicting renal function decline.

**Subjects/Methods:**

In total, 5438 Japanese urban residents (median age 48 years) who participated in a public health screening program for 4 consecutive years were enrolled. Systemic arterial stiffness was assessed by cardio-ankle vascular index (CAVI). The predictability of the new-onset renal function decline (eGFR < 60 mL/min/1.73 m^2^) by replacing high WC with high ABSI (ABSI ≥ 0.080) was examined using three sets of MetS diagnostic criteria: Japanese, IDF and NCEP-ATPIII.

**Results:**

In Japanese and NCEP-ATPIII criteria, MetS diagnosed using ABSI (ABSI-MetS) was associated with significantly higher age-adjusted CAVI compared to non-MetS, whereas MetS diagnosed using WC (WC-MetS) showed no association. Kaplan–Meier analysis of the rate of new-onset renal function decline over 4 years (total 8.7%) showed remarkable higher rate in subjects with ABSI-MetS than in those without (log-rank test *p* < 0.001), but almost no difference between subjects with and without WC-MetS (*p* = 0.014–0.617). In gender-specific Cox-proportional hazards analyses including age, proteinuria, and treatments of metabolic disorders as confounders, ABSI-MetS (Japanese criteria for both sexes, IDF criteria for men) contributed independently to the new-onset renal function decline. Of these, the contribution of IDF ABSI-MetS disappeared after adjustment by high CAVI in the subsequent analysis.

**Conclusion:**

In this study, replacing WC with ABSI in MetS diagnostic criteria more efficiently predicted subjects at risk of renal function decline and arterial stiffening.

## Introduction

Abdominal obesity with visceral fat accumulation has been considered to play an essential role in the development of cardiometabolic disorders including glucose intolerance, dyslipidemia and hypertension, so-called the metabolic syndrome (MetS) [1, 2]. Several epidemiological studies have demonstrated that MetS increases the risk of not only cardiovascular disease (CVD) morbidity and mortality [3, 4], but also the incidence of chronic kidney disease (CKD) [5, 6] and end-stage renal disease (ESRD) [7]. On the other hand, the validity of MetS diagnostic criteria remains controversial. Reaven [8] highlighted the fact that there are many non-MetS patients who are clearly at higher risk of CVD than MetS patients. Furthermore, it has been claimed that MetS does not necessarily predict CVD risk above and beyond its individual components [9, 10]. Some reports have also shown that elevated waist circumference (WC), an indicator of abdominal obesity for diagnosing MetS, is associated with CKD to a less extent after adjusting for MetS components [11, 12]. We thus hypothesized that these incongruous phenomena may be attributed to the uncertainty of WC as an indicator of visceral fat accumulation or the definition of MetS.

Increase in body mass index (BMI) may be positively related to improved vascular function assessed by flow-mediated dilation [13] and cardio-ankle vascular index (CAVI) [14], even though elevated body weight contributes to an increased incidence of obesity-related metabolic disorders. In other words, BMI is not necessarily suitable for efficient stratification of CVD risks, because of the obesity paradox in which an increase in BMI may potentially reflect vasoprotective body composition (such as subcutaneous and/or skeletal muscle tissue). Partially because of this situation, abdominal obesity, the basic risk factor of MetS, is evaluated by WC in all the diagnostic criteria for MetS. However, since WC correlates closely with BMI, it is difficult to distinguish between the two as epidemiological risk factors [15, 16]. In fact, MetS diagnosed by the current criteria is not necessarily associated with systemic arterial stiffening assessed by CAVI [17–19].

Since the evaluation of visceral fat accumulation by computed tomography (CT) is difficult in routine clinical practice, many abdominal obesity indices using anthropometric measurements have been established. “A Body Shape Index (ABSI)” is an abdominal obesity index that is independent of BMI [16, 20], and is calculated by dividing WC by an allometric regression of weight and height [21]. In addition, ABSI reflects visceral fat accumulation well [22, 23], and is suitable in screening subjects with CVD risks [24] and predicting cardiovascular mortality [25]. We have reported that increased ABSI reflects not only the severity of metabolic disorders, but also systemic arterial stiffening indicated by high CAVI in Japanese general population [16].

With the above background, we consider that there is room for improvement of the current diagnostic criteria for MetS. In the present study, we evaluated systemic arterial stiffness in MetS patients diagnosed using ABSI instead of WC. We also examined whether MetS diagnosed using ABSI predicts the new-onset renal function decline.

## Materials and methods

### Subjects and design

We performed a retrospective cohort study in Japanese urban residents who underwent health screening between January 2013 and December 2018.

### Data collection

The population-based sample used in the present analysis comprised 144,876 Japanese subjects residing in major cities nationwide, who participated in the annual CVD and cancer screening program organized by the Japan Health Promotion Foundation. Participants were volunteers who were not paid and were not recruited for this study (unlike subjects of a clinical trial). Subjects without four consecutive years of data (139,438) or sufficient data (1,534) were excluded, and a total of 5438 subjects were finally enrolled in the present study.

All parameters were assessed using standardized methods. Height and body weight (BW) were measured, and BMI was calculated as follows: BW (kg) divided by square of height (m). WC (m) was measured horizontally at the height of the umbilicus, with the participant standing and arms hanging relaxed. ABSI was calculated by the following formula: ABSI = WC/(BMI^2/3^ × height^1/2^) [21]. Currently, an online calculator implementing the derived formula for anthropometric risk indicators including ABSI is freely available at https://nirkrakauer.net/sw/ari-calculator.html.

Blood samples were collected from the antecubital vein in the morning after 12 h of fasting to measure fasting plasma glucose (FPG), triglycerides (TG) and high-density lipoprotein cholesterol (HDL-C). Low-density lipoprotein-cholesterol (LDL-C) was calculated using Friedewald formula: LDL-C = total cholesterol (TC) – (HDL-C) – (TG/5). Since this formula is not valid for patients with TG ≥ 400 mg/dL (4.52 mmol/L), subjects with baseline TG ≥ 4.52 mmol/L (0.6% of all participants) were excluded from the analysis of LDL-C. Hyper-LDL cholesterolemia was defined as LDL-C ≥ 3.62 mmol/L [26], and the determination of other cardiometabolic risks including hypertension, impaired fasting glucose, hypertriglyceridemia and hypo-HDL cholesterolemia followed the Japanese MetS diagnostic criteria described below.

The estimated glomerular filtration rate (eGFR) was calculated by the following equation from the Japanese Society of Nephrology [27]:

$${{{{{\mathrm{eGFR}}}}}}\left( {{{{{{\mathrm{mL/min/}}}}}}1.73\,m^2} \right) = 194 \times {{{{{\mathrm{creatinine}}}}}}^{ - 1.094} \times {{{{{\mathrm{age}}}}}}^{ - 0.287}\left( { \times 0.739\,{{{{{\mathrm{if}}}}}}\,{{{{{\mathrm{female}}}}}}} \right).$$

In addition, renal function decline was defined as eGFR <60 mL/min/1.73 m^2^, corresponding to GFR category 3a or worse [28].

The collected spot urine samples were used for urinalysis by dipstick method. Urinalysis results were recorded as (−), (±), (1+), (2+), and (3+). Proteinuria was defined as (1+) or more, which corresponded to a urine protein level of 30 mg/dl or more.

### Measurement of CAVI and blood pressure

Arterial stiffness was assessed by CAVI using VaSera VS-1500 (Fukuda Denshi Co Ltd, Tokyo, Japan). CAVI was calculated according to the following formula: CAVI=a{(2ρ/ΔP)×ln(Ps/Pd)PWV^2^}+b, where Ps is systolic blood pressure (SBP); Pd is diastolic blood pressure (DBP); ΔP is Ps - Pd; ρ is blood density; PWV is cardio-ankle pulse wave velocity, and a and b are constants.

The cuffs were wrapped around the upper arms and ankles of subjects in spine position with the head in the midline position. Examination was started after 5 min of rest. When detecting pulse waves in the upper arms and ankles with cuffs, a low cuff pressure of 30–50 mmHg was used to minimize the influence of cuff pressure on hemodynamics. BP was measured from the upper arm cuffs. PWV was determined by dividing the arterial length by the time taken for the pulse wave to propagate from the aortic valve to the ankle, and was measured using the upper arm and ankle cuffs. Because CAVI may be falsely low in patients with severe arterial occlusive disease, we excluded subjects with an ankle-brachial index lower than 0.90. The details of CAVI have been reported previously [29]. We arbitrarily defined “high CAVI” as equal to or higher than 9.0 in all participants, corresponding substantially to the cut-off for the presence of coronary artery stenosis [30, 31].

### Criteria of metabolic syndrome and our proposal

In the present study, we tested the following three sets of diagnostic criteria for MetS: the criteria developed by the Japanese Committee for the Diagnostic Criteria of MetS (Japanese criteria) [32], the National Cholesterol Education Program–Adult Treatment Panel III (NCEP-ATPIII) criteria [33] and the International Diabetes Federation (IDF) criteria [34].

In Japanese criteria, MetS is defined as the presence of at least two of the following three abnormalities: [1] TG ≥ 150 mg/dL (1.69 mmol/L) and/or HDL-C < 40 mg/dL (1.03 mmol/L); [2] SBP ≥ 130 mmHg and/or DBP ≥ 85 mmHg; [3] FPG ≥ 110 mg/dL (6.11 mmol/L), in the presence of high WC (≥85 cm in men, and ≥ 90 cm in women).

In IDF criteria, MetS is defined as the presence of at least two of the four abnormalities: [1] TG ≥ 150 mg/dL (1.69 mmol/L); [2] HDL-C < 40 mg/dL (1.03 mmol/L); [3] SBP ≥ 130 mmHg and/or DBP ≥ 85 mmHg [4] FPG ≥ 110 mg/dL (6.11 mmol/L), in the presence of high WC (≥90 cm in men, and ≥80 cm in women).

In NCEP-ATPIII criteria, MetS is defined as the presence of at least three of the five abnormalities: [1] TG ≥ 150 mg/dL (1.69 mmol/L); [2] HDL-C < 40 mg/dL (1.03 mmol/L); [3] SBP ≥ 130 mmHg and/or DBP ≥ 85 mmHg; [4] FPG ≥ 100 mg/dL (5.55 mmol/L); [5] WC ≥ 102 cm in men, and WC ≥ 88 cm in women.

Initially, the FPG threshold in NCEP-ATPIII was 6.11 mmol/L. Later, however, the American Diabetes Association lowered the FPG threshold used to identify individuals with impaired fasting glycemia (IFG) from 6.11 mmol/L to 5.55 mmol/L, and the NCEP-ATPIII followed suit [35].

For all three sets of diagnostic criteria, treatments for dyslipidemia, hypertension and diabetes were counted as positive for the respective abnormalities. In the IDF/NCEP-ATP III criteria, hypo-HDL-cholesterolemia was treated as an independent factor different from treatments for dyslipidemia.

In our previous cross-sectional study enrolling 62,514 healthy Japanese subjects, the cut-off value of ABSI in predicting high CAVI, which we estimated by Youden index on receiver operating characteristics (ROC) curve, was 0.080 irrespective of gender [16]. Based on this finding, we adopted a proposal to replace high WC with high ABSI (≥0.080, both sexes) as the criterion of abdominal obesity in the present study. Additionally, we also examined whether there is a difference in the prediction of the new-onset renal function decline between MetS diagnosed conventionally using WC (WC-MetS) and MetS diagnosed using ABSI (ABSI-MetS).

### Statistical analysis

The SPSS software (version 27.0.1, Chicago, IL, USA) was used for statistical analyses. All data are expressed as median [interquartile range (IQR)] or mean ± standard deviation. Mann-Whitney *U* test or Fisher’s exact test was performed to examine gender difference in clinical variables or prevalence of CVD risk factors. One way analysis of covariance with the covariate set to age followed by Bonferroni multiple comparison test were used to compare age-adjusted CAVI between subjects with and those without WC-MetS or ABSI-MetS by gender. Kaplan–Meier survival analysis was employed to estimate the time to end point, and log-rank test was used to compare between two groups. Cox-proportional hazards analysis was performed to identify the contribution of variables to the new-onset renal function decline, and the result is expressed as hazard ratio (HR) with 95% confidence interval (CI). In all comparisons, two-sided *p* values < 0.05 were considered statistically significant.

## Results

### Baseline clinical and biochemical characteristics of participants

In this retrospective cohort study, a total of 5,438 Japanese urban residents (2368 men and 3070 women, median age 48 years, median BMI 21.9 kg/m^2^ at baseline) who were examined in 4 consecutive annual health checks were studied. Table 1 compares the baseline clinical characteristics of subjects with and without Japanese WC-/ABSI-MetS at the first examination.

**Table 1:** Baseline clinical and biochemical characteristics of participants.

Variables	Total	Japanese WC-MetS		Japanese ABSI-MetS	
		No	Yes	No	Yes
Number (Male, %)	5438 (43.5)	5,157 (41.7)	281 (78.3)^*^	5,021 (44.6)	417 (30.9)^*^
Age (years)	48 (40–58)	48 (40-58)	54 (45–63)^*^	47 (40-57)	64 (55–70)^*^
Age ≥ 65 years (%)	764 (14.0)	704 (13.7)	60 (21.4)^†^	568 (11.3)	196 (47.0)^†^
Current smoking (%)	733 (13.5)	679 (13.2)	5(19.2)^†^	695 (13.8)	38 (9.1)^†^
Habitual alcohol drinking (%)	847 (15.6)	783 (15.2)	(22.8)^†^	784 (15.6)	63 (15.1)
BMI (kg/m2)	21.9 (20.0–23.9)	21.7 (19.9–23.6)	26.6 (25.2–28.8)^*^	21.9 (19.9–23.9)	22.3 (20.6–24.3)^*^
BMI ≥ 25 kg/m^2^ (%)	958 (17.6)	737 (14.3)	221 (78.6)^†^	873 (17.4)	85 (20.4)
WC (meter)	0.790 (0.728–0.849)	0.784 (0.725–0.840)	0.918 (0.880–0.965)^*^	0.785 (0.723–0.845)	0.837 (0.793–0.883)^*^
WC ≥ 85 (m) or 90 (f) cm (%)	1,059 (19.5)	0 (0.0)	281 (100.0)^†^	955 (19.0)	104 (24.9)^†^
WC ≥ 90 (m) or 80 (f) cm (%)	1397 (25.7)	1225 (23.8)	172 (61.2)^†^	1173 (23.4)	224 (53.7)^†^
WC ≥ 102 (m) or 88 (f) cm (%)	356 (6.5)	272 (5.3)	84 (29.9)^†^	278 (5.5)	78 (18.7)^†^
ABSI	0.0785 (0.0758–0.0814)	0.0785 (0.0758–0.0814)	0.0789 (0.0769–0.0819)	0.0781 (0.0756–0.0807)	0.0832 (0.0814–0.0856)^*^
ABSI ≥ 0.080 (%)	1,967 (36.2)	1863 (36.1)	104 (37.0)^*^	1,550 (30.9)	417 (100.0)^†^
SBP (mmHg)	115 (107–126)	114 (106–125)	135 (126–143)^*^	114 (106–124)	136 (129–144)^*^
DBP (mmHg)	72 (66–80)	72 (65–79)	85 (79–92)^*^	71 (65–79)	83 (76–88)^*^
SBP ≥ 130 and/or	1287 (23.7)	1067 (20.7)	220 (78.3)^†^	952 (19.0)	335 (80.3)^†^
DBP ≥ 85 mmHg (%)					
Receiving hypertension treatment (%)	499 (9.2)	396 (7.7)	103 (36.7)^†^	348 (6.9)	151 (36.2)^†^
CAVI	7.6 (7.1–8.3)	7.6 (7.1-8.3)	7.9 (7.4-8.7)^*^	7.6 (7.1-8.2)	8.7 (8.0-9.3)^*^
CAVI ≥ 9.0 (%)	631 (11.6)	577 (11.2)	54 (19.2)^†^	466 (9.3)	165 (39.6)^†^
FPG (mmol/L)	4.83 (4.50–5.22)	4.83 (4.50–5.22)	5.05 (4.66–5.55)^*^	4.77 (4.50–5.16)	5.11 (4.77–5.72)^*^
FPG > 5.55 mmol/L (%)	1735 (31.9)	1638 (31.8)	97 (34.5)	1534 (30.6)	201 (48.2)^†^
FPG > 6.11 mmol/L (%)	114 (2.1)	86 (1.7)	28 (10.0)^†^	75 (1.5)	39 (9.4)^†^
Receiving diabetes treatment (%)	80 (1.5)	62 (1.2)	18 (6.4)^†^	58 (1.2)	22 (5.3)^†^
LDL-C (mmol/L)	3.23 (2.72–3.80)	3.21 (2.72–3.75)	3.78 (3.15–4.19)^*^	3.18 (2.69–3.72)	3.80 (3.13–4.22)^*^
LDL-C ≥ 3.62 mmol//L (%)	1761 (32.4)	1582 (30.7)	179 (63.7)^†^	1489 (29.7)	272 (65.2)^†^
HDL-C (mmol/L)	1.81 (1.50–2.15)	1.84 (1.53–2.17)	1.55 (1.29–1.78)^*^	1.81 (1.50–2.15)	1.73 (1.47–2.04)^*^
TG (mmol/L)	0.87 (0.63–1.25)	0.85 (0.62–1.21)	1.34 (1.04–1.87)^*^	0.85 (0.62–1.23)	1.11 (0.84–1.46)^*^
TG ≥ 1.69 and/or	665 (12.2)	578 (11.2)	87 (31.0)^†^	586 (11.7)	79 (18.9)^†^
HDL-C < 1.03 mmol/L (%)					
Receiving dyslipidemia treatment (%)	404 (7.4)	344 (6.7)	60 (21.4)^†^	282 (5.6)	122 (29.3)^†^
Creatinine (mg/dL)	0.71 (0.60–0.84)	0.70 (0.60–0.83)	0.83 (0.73–0.93)^*^	0.71 (0.61–0.85)	0.67 (0.59–0.80)^*^
eGFR (mL/min/1.73m^2^)	78.2 (69.6–87.8)	78.4 (69.7–88.0)	74.4 (63.9–84.3)^*^	78.7 (70.0–88.1)	72.7 (64.1–82.4)^*^
eGFR < 60 mL/min/1.73m^2^ (%)	395 (7.3)	349 (6.8)	46 (16.4)^†^	323 (6.4)	72 (17.3)^†^
Proteinuria (%)	283 (5.2)	250 (4.8)	33 (11.7)^†^	258 (5.1)	25 (6.0)
IDF WC-MetS (%)	366 (6.7)	268 (5.2)	98 (34.9)^†^	232 (4.6)	134 (32.1)^†^
IDF ABSI-MetS (%)	504 (9.3)	348 (6.7)	40 (14.2)^†^	150 (3.0)	238 (57.1)^†^
NCEP ATPIII WC-MetS (%)	222 (4.1)	150 (2.9)	72 (25.6)^†^	136 (2.7)	86 (20.6)^†^
NCEP ATPIII ABSI-MetS (%)	614 (11.3)	394 (7.6)	73 (26.0)^†^	227 (4.5)	240 (57.6)^†^

Data are presented as median (IQR). *p < 0.001, Mann–Whitney U test and †*p* < 0.05, Fisher’s exact test, compared to subjects without MetS.

*BMI* body mass index, *WC* weight circumference, *ABSI* a body shape index, *SBP* systolic blood pressure, *DBP* diastolic blood pressure, *CAVI* cardio-ankle vascular index, *FPG* fasting plasma glucose, *LDL-C* low-density lipoprotein-cholesterol, *HDL-C* high-density lipoprotein-cholesterol, *TG* triglyceride, *eGFR* estimated glomerular filtration rate, *WC-MetS* conventional metabolic syndrome (MetS) diagnosed using waist circumference (WC), *ABSI-MetS* MetS diagnosed using a body shape index (ABSI) instead of WC; Japanese, criteria developed by the Japanese Committee for the Diagnostic Criteria of MetS, *IDF* International Diabetes Federation, *NCEP-ATPIII* National Cholesterol Education Program Adult Treatment Panel III, *IQR* interquartile range.

Subjects with the two types of MetS commonly showed higher age, BMI, WC, BP, CAVI, FPG, LDL-C, TG, and creatinine, and lower HDL-C compared to those without.

The prevalence of WC-MetS and ABSI-MetS diagnosed by IDF and NCEP-ATPIII (IDF WC-MetS, IDF ABSI-MetS, NCEP-ATPIII WC-MetS and NCEP ATPIII ABSI-MetS) was also higher in subjects with Japanese WC/ABSI-MetS.

Most of the subjects with Japanese WC-MetS were male, whereas most of the subjects with Japanese ABSI-MetS were female. Only subjects with Japanese WC-MetS had a higher prevalence of current smoking, habitual alcohol drinking and proteinuria. On the other hand, only Japanese subjects with ABSI-MetS showed higher ABSI.

### Age-adjusted CAVI according to MetS criteria defined using WC or ABSI

**Fig. 1:**
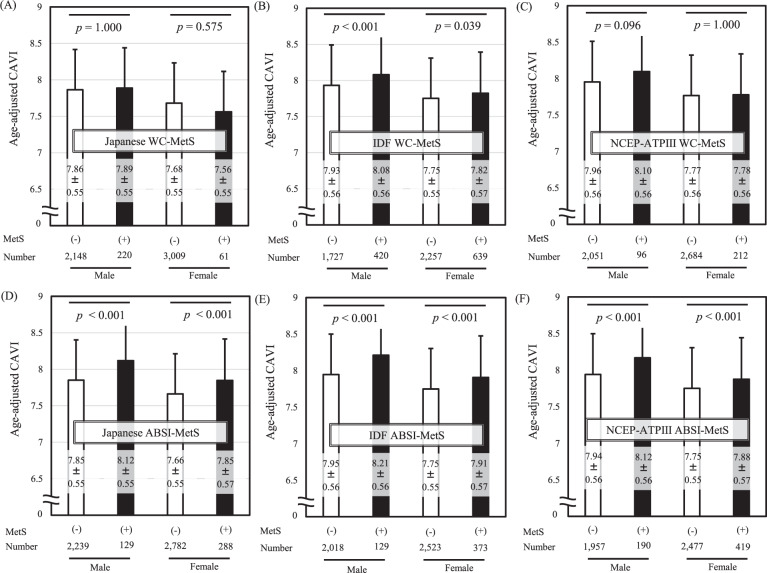
Comparison of age-adjusted CAVI in MetS (+) vs. MetS (-) diagnosed by various MetS criteria using WC or ABSI. MetS was diagnosed by (**A**) Japanese, (**B**) IDF and (**C**) NCEP-ATPIII criteria using WC, and also diagnosed by (**D**) Japanese, (**E**) IDF and (**F**) NCEP-ATPIII criteria using ABSI instead of WC. Data are presented as mean ± standard deviation and analyzed by One way analysis of covariance with the covariate set to age followed by Bonferroni multiple comparison tests. CAVI cardio-ankle vascular index, WC-MetS conventional metabolic syndrome (MetS) diagnosed using waist circumference (WC); ABSI-MetS MetS diagnosed using a body shape index (ABSI) instead of WC; Japanese, criteria developed by the Japanese Committee for the Diagnostic Criteria of MetS, IDF International Diabetes Federation, NCEP-ATPIII National Cholesterol Education Program Adult Treatment Panel III.

The differences in age-adjusted CAVI between MetS and non-MetS subjects diagnosed by various MetS criteria using WC or ABSI are shown in Fig. 1. Age-adjusted CAVI did not differ significantly between subjects diagnosed with Japanese WC-MetS or NCEP-ATPIII WC-MetS compared to those without the diagnosis (Fig. 1A, C). In contrast, age-adjusted CAVI was clearly higher in subjects diagnosed with Japanese ABSI-MetS or NCEP-ATPIII ABSI-MetS compared to those without the diagnosis (Fig. 1D, F). For IDF criteria only, IDF-MetS subjects had higher age-adjusted CAVI than non-IDF-MetS subjects when diagnosed using either WC or ABSI (Fig. 1B, E). The same results were observed in men and women.

### Baseline clinical and biochemical characteristics in subjects with or without renal function decline during 4-year study period

In this analysis, 395 (7.3%) subjects with eGFR lower than 60 mL/min/1.73 m^2^ at baseline were excluded out of 5,438 total participants. And then, 474 (8.7%) developed the new-onset renal function decline during the 4-year study period. We then investigated the baseline characteristics between subjects with and those without new-onset renal function decline (Table S1). The group with renal function decline had significantly higher male ratio, age, ABSI, BP and CAVI; and lower HLD-C, LDL-C and eGFR compared to the group without renal function decline. There were no significant differences in current smoking rate, WC, BMI, FPG, and TG between the two groups. HDL-C, LDL-C and eGFR were rather low in subjects who developed renal function decline. When diagnosed with Japanese or NCEP-ATPIII criteria, the prevalence of ABSI-MetS was markedly higher in the group with renal function decline than in the group without renal function decline, while there was no difference in the prevalence of WC-MetS between two groups. On the other hand, when diagnosed with IDF criteria, the prevalence of both WC-MetS and ABSI- Mets was significantly higher in the group with renal function decline compared to the group without renal function decline.

### Kaplan–Meier curves for renal function decline in MetS and non-MetS subjects diagnosed by different MetS criteria using WC or ABSI

**Fig. 2:**
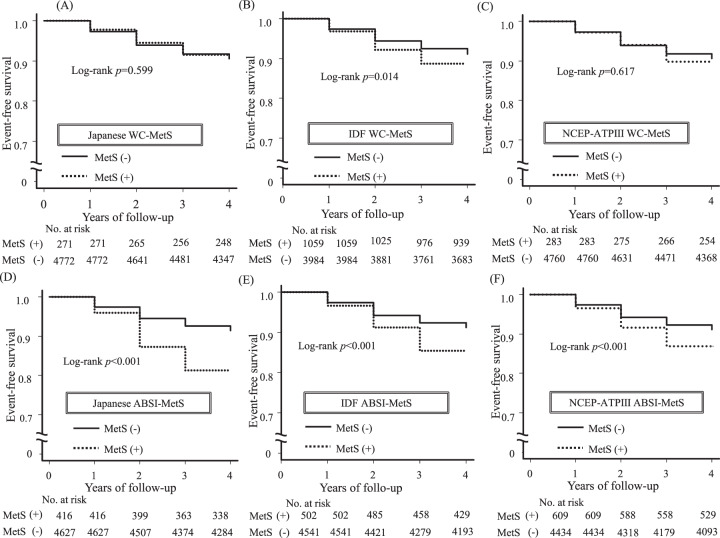
Kaplan-Meier curves for the rate of renal function decline in MetS (+) and MetS (–) diagnosed by various MetS criteria using WC or ABSI. MetS was diagnosed by (**A**) Japanese, (**B**) IDF and (**C**) NCEP-ATPIII criteria using WC, and also diagnosed by (**D**) Japanese, (**E**) IDF and (**F**) NCEP-ATPIII criteria using ABSI instead of WC. WC-MetS, conventional metabolic syndrome (MetS) diagnosed using waist circumference (WC); ABSI-MetS, MetS diagnosed using a body shape index (ABSI) instead of WC; Japanese, criteria developed by the Japanese Committee for the Diagnostic Criteria of MetS; IDF International Diabetes Federation, NCEP-ATPIII National Cholesterol Education Program Adult Treatment Panel III.

Kaplan–Meier survival analyses for the new-onset renal function decline when MetS was diagnosed by various criteria are shown in Fig. 2. The survival rates and 95% CIs were described in Table S2.

When using ABSI in MetS diagnosis, the cumulative rate of renal function decline occurring over 4 years was significantly higher in ABSI-MetS subjects than in non- ABSI-MetS subjects diagnosed by Japanese, IDF and NCEP-ATPIII criteria (Fig. 2D–F). On the other hand, when using WC in MetS diagnosis, the cumulative rates of renal function decline were comparable in WC-MetS and non-WC-MetS subjects diagnosed by Japanese and NCEP-ATPIII criteria (Fig. 2A, C), although the rate of renal function decline was significantly higher in IDF WC-MetS subjects than in non-IDF WC-MetS subjects (Fig. 2B), similar to the result of IDF ABSI-MetS (Fig. 2E).

As mentioned earlier, the cut-off value of ABSI in predicting high CAVI adopted in this study was 0.080, regardless of gender. On the other hand, the cut-off value of baseline ABSI in predicting renal function decline newly occurring during the 4-year study period observed in this study (474 out of 5,438 total participants, 8.7%) was 0.079 as estimated by Youden index on ROC curve analysis (AUC = 0.584, *p* < 0.001).

### Cox-proportional hazards models for the association of the new-onset renal function decline with Japanese MetS and clinical variables

Next, the contribution of WC-MetS and ABSI-MetS diagnosed by Japanese criteria to the new-onset renal function decline was examined using Cox-proportional hazards analyses (Table 2). Since aging, high CAVI, proteinuria and therapeutic intervention for metabolic disorders are generally the main factors influencing renal function, they were also used as confounders in the analyses. Age (every 1 year) was included in the Cox model as a stratifying variable.

**Table 2:** Cox-proportional hazards models for the association of the new-onset renal function decline with Japanese MetS and clinical variables.

Variables	Age (every 1 y)	Male gender	CAVI ≥ 9.0	Proteinuria	Receiving diabetes treatment	Receiving hypertension treatment	Receiving dyslipidemia treatment	Japanese WC-MetS	Japanese ABSI-MetS
Model 1	1.019 (1.009–1.029)	1.277 (1.067–1.529)^*^	3.415 (2.661–4.383)^*^	0.984 (0.669–1.446)	0.971 (0.565–1.668)	1.459 (1.154–1.845)^*^	1.173 (0.901–1.529)	0.718 (0.487–1.057)	–
Model 2	1.018 (1.007–1.028)^*^	1.307 (1.092–1.565)^*^	3.246 (2.530–4.166)^*^	0.939 (0.638–1.380)	0.891 (0.518–1.532)	1.314 (1.038–1.662)^*^	1.030 (0.785–1.352)	–	1.623 (1.273–2.069)^*^

^*^p < 0.05, Hazards ratios (95% confidence intervals). Renal function decline is defined as eGFR<60 mL/min/1.73m^2^ during the 4-year study period. All confounders are included in the table.

WC-MetS, conventional metabolic syndrome (MetS) diagnosed using waist circumference (WC); ABSI-MetS, MetS diagnosed using a body shape index (ABSI) instead of WC; Japanese, criteria developed by the Japanese Committee for the Diagnostic Criteria of MetS.

Japanese ABSI-MetS was an independent contributor to the new-onset renal function decline (Model 2), whereas Japanese WC-MetS was not (Model 1). In both models, aging, male gender, high CAVI and treatment of hypertension were also the contributors, but proteinuria, and treatment of diabetes and dyslipidemia were not.

### Hazard ratios (95% confidence intervals) for the association of the new-onset renal function decline with MetS defined by three types of definitions

Finally, we performed Cox-proportional hazards analyses by gender to examine the contribution of three different definitions of MetS associated with new-onset renal function decline (Table 3). ABSI-MetS was associated with an increase in CAVI as shown in Fig. 1. On the other hand, as shown in Table 2, high CAVI contributed to the new-onset renal function decline independently of Japanese ABSI-MetS. Therefore, we also investigated whether the contribution of each MetS to renal function decline varied according to the presence or absence of high CAVI. The 1st analysis did not include high CAVI as a confounder, but the 2nd analysis included. Furthermore, as in Table 2, proteinuria, receiving treatments for diabetes, hypertension and dyslipidemia were used as confounders.

**Table 3:** Hazard ratios (95% confidence intervals) for the association of the new-onset renal function decline with MetS defined by three types of definitions.

Variables		Japanese WC-MetS	Japanese ABSI-MetS	IDF WC-MetS	IDF ABSI-MetS	NCEP-ATPIII WC-MetS	NCEP-ATPIII ABSI-MetS
Male	1st analysis	0.665 (0.419–1.056)	1.859 (1.266–2.730)^*^	1.092 (0.793–1.505)	1.598 (1.069–2.390)^*^	0.729 (0.390–1.365)	1.269 (0.858–1.878)
	2nd analysis	0.697 (0.440–1.102)	1.534 (1.038–2.265)^*^	0.943 (0.684–1.302)	1.254 (0.834–1.886)	0.664 (0.357–1.237)	1.043 (0.704–1.546)
Female	1st analysis	0.756 (0.368–1.553)	1.611 (1.172–2.214)^*^	0.782 (0.562–1.087)	1.147 (0.833–1.579)	0.829 (0.508–1.355)	1.083 (0.786–1.492)
	2nd analysis	0.881 (0.430–1.805)	1.463 (1.063–2.014)^*^	0.728 (0.523–1.014)	1.026 (0.745–1.414)	0.828 (0.549–1.250)	0.985 (0.715) (1.356)

^*^p < 0.05, Cox-proportional hazards analyses. Renal function decline is defined as eGFR <60 mL/min/1.73 m^2^ during the 4-year study period.

The hazard ratios for each MetS are expressed after adjustment for the following confounders;

1st analysis: adjusting for age (every 1 y), proteinuria, and receiving treatments for diabetes, hypertension and dyslipidemia.

2nd analysis: adjusting for confounders using in Model 1 and CAVI ≥ 9.0.

WC-MetS, conventional metabolic syndrome (MetS) diagnosed using waist circumference (WC); ABSI-MetS, MetS diagnosed using a body shape index (ABSI) instead of WC; Japanese, criteria developed by the Japanese Committee for the Diagnostic Criteria of MetS; IDF, International Diabetes Federation; NCEP-ATPIII; National Cholesterol Education Program Adult Treatment Panel III.

The results showed that Japanese ABSI-MetS independently contributed to the new-onset renal function decline in both genders with and without high CAVI, whereas most MetS diagnosed by other criteria did not contribute. IDF ABSI-MetS in male also contributed to the new-onset renal function decline in the 1st analysis, but the contribution disappeared after adjustment by high CAVI in the 2nd analysis.

## Discussion

This retrospective cohort study conducted in real-world Japanese population verified whether there is a difference between using conventional WC and replacing WC with ABSI in three difference sets of MetS diagnostic criteria in predicting the degree of systemic arterial stiffness and the new-onset renal function decline.

Compared to WC-MetS, ABSI-MetS was strongly associated with increased CAVI in both genders. Additionally, subjects in whom renal function decline occurred during the 4-year study period had higher prevalence of ABSI-MetS at baseline compared to those without renal function decline. Kaplan-Meier analyses revealed almost no difference in the rate of renal function decline between subjects with and those without WC-MetS when diagnosed by Japanese and NCEP-ATPIII criteria, whereas significantly increased rates of renal function decline were observed in subjects with ABSI-MetS compared to those without when diagnosed by all three sets of criteria. In Cox-proportional hazards analyses, aging, male gender, high CAVI, hypertension treatment and Japanese ABSI-MetS, but not WC-MetS, were independent contributors to the new-onset renal function decline. IDF ABSI-MetS also showed the contribution to the new-onset renal function decline, if high CAVI is not included as a confounder in the Cox-proportional hazards analysis in male. The strength of this study is to demonstrate that replacing WC with ABSI in MetS diagnosis more efficiently predicts people at risk of occurring renal function decline in large-scale health screening.

Cardio-ankle vascular index (CAVI) is known to reflect the stiffness of the arterial tree from the origin of the aorta to the ankle, independent of BP at the time of measurement [29, 31], and has been reported to be associated positively with a number of CVD risks [36, 37], severity of CVD [30], and future CVD events [38]. Furthermore, CAVI was not only associated with renal function in cross-sectional studies [39], but also predicted renal function decline in retrospective cohort studies [40, 41]. Likewise, high CAVI predicted the new-onset renal function decline also in the present study. High CAVI reflects not only organic lesion of the macro vessel, but also microcirculatory disorders induced by inflammation and oxidative stress [31, 40]. Accordingly, vascular ageing, indicated by high CAVI, may lead to future renal function decline via damage in the microvasculature. On the other hand, we have reported that BMI is inversely related to CAVI [14], even though elevated BMI contributes to the development of obesity-related metabolic disorders. Considering that high CAVI correlates positively with abdominal obesity index [16] and that visceral fat reduction is directly related to reduced CAVI [42], the validity of using BMI and BMI-dependent abdominal obesity indices to accurately reflect body composition that leads to vascular toxicity is questionable. Accordingly, a suitable abdominal obesity index for use in MetS diagnosis should reflect vascular toxicity due to visceral fat accumulation, and should be independent of BMI. ABSI seems to be an appropriate index that fulfills these requirements [16]. The finding of the present study that ABSI-MetS predicts the new-onset renal function decline may support the appropriateness of ABSI as an index of abdominal obesity. However, we conveniently adopted the cut-off value of ABSI based on the results of ROC analysis for high CAVI using cross-sectional data. Further investigation is needed to determine the appropriate cut-off value of ABSI to predict CVD morbidity and mortality.

In the Japanese MetS criteria, as mentioned earlier, high WC was ≥85 cm in men, and ≥90 cm in women [32], and these are Japanese limited cut-offs corresponding to visceral fat area (VFA) of 100 cm^2^ measured by CT [43]. It is often pointed out that these cut-offs of WC deviate from non-Japanese MetS criteria. Since WC is epidemiologically nearly identical to BMI and does not necessarily reflect VFA, it may not be appropriate to calculate its cut-off from the regression line between VFA and WC. On the other hand, the extent to which ABSI of 0.080 is associated with VFA awaits further verification.

In this study, ABSI-MetS was associated with increased CAVI, and the adjustment for high CAVI in the Cox-proportional hazards analysis weaken the contribution of IDF ABSI-MetS to the new-onset renal function decline (2^nd^ analysis in Table 3). These findings are consistent with the fact that high ABSI is related to high CAVI [16]. On the other hand, ABSI-MetS and high CAVI contributed independently to the new-onset renal function decline (Table 2), indicating that CAVI may also be affected by factors other than abdominal obesity-related metabolic disorders [31]. Meanwhile, why might high ABSI and high CAVI simultaneously contribute to renal function decline? One reason may be that abdominal obesity, which induces arterial stiffening indicated by increased CAVI, may also be involved in renal function decline via obesity-related glomerulopathy (ORG). It has been reported that obesity is not only the underlying pathology of metabolic disorders, but also an independent risk of ESRD even after adjustment for atherosclerotic risk factors [43]. ORG is defined pathologically as glomerulomegaly with focal segmental glomerulosclerosis [44], and the severity depends on body fat accumulation [45]. In patients with ORG, various extra- and intra-renal pathophysiologies are involved in the progression of CKD [46] for the reasons given below. First, pro-inflammatory adipocytokines derived from extra-renal visceral adipose tissue contribute to the pathogenesis of ORG [47]. Second, the role of intra-renal adipose tissue in MetS patients has also been reported [48]. An increase in ectopic adipose tissue in the renal sinuses can alter physical forces by restricting the outflow of blood and lymph fluid from the kidneys, which may promote sodium reabsorption and focal arteriosclerosis [49]. Considering that ABSI reflects so-called “obesity-related” metabolic disorders regardless of obesity [24], it is possible that ABSI-MetS predicted ORG even in the present study population, which mainly included non-overweight individuals.

The reason why NCEP-ATP III ABSI-MetS did not independently contribute to the new-onset renal function decline in both men and women is unclear (Table 3). However, this result may suggest the superiority of treating hypertriglyceridemia and low-HDL cholesterol as a single risk factor, in addition to the significance to recognize abdominal obesity as an essential requirement for MetS diagnosis.

Sato et al. previously reported the predictability of ABSI for all-cause mortality in Japanese population [50]. In the result, ABSI could be applied to predict all-cause mortality in men, but it shows only weak association with all-cause mortality in women, especially in the presence of chronic kidney disease (CKD). Furthermore, the predictability of ABSI for all-cause mortality in men was independent of the presence or absence of CKD. To begin with, there was little difference in ABSI between those with and without CKD in both genders. These findings do not seem to be consistent with our results showing that increased ABSI impairs renal function. However, ABSI may have better predictability for mortality and morbidity in Caucasians than in Asians [50]. Whether or not the risk of all-cause mortality enhanced by ABSI is partially mediated by renal function decline in the Japanese population awaits further investigation [51].

The risks of progression to kidney failure associated with a given level of eGFR are independently increased in patients with higher levels of proteinuria [52]. However, proteinuria was not extracted as a contributor to the new-onset renal function decline in this study. In addition, at baseline, subjects with ABSI-MetS did not necessarily have a higher prevalence of proteinuria. High specific gravity and hematuria may lead to false positives for proteinuria by urinalysis [53]. In addition to the low prevalence of impaired glucose tolerance, which is the main cause of proteinuria, the fact that the subjects were fasting for more than 12 hours and that many of them were premenopausal women may have influenced the results of urinalysis.

One limitation of this study is that our findings may not be generalized to other ethnic groups. Further research is needed to address this issue and to confirm whether therapeutic approaches to reduce ABSI decrease the risk of developing ESRD and CVD.

## Conclusion

Replacing WC with ABSI in MetS diagnostic criteria may more efficiently predict people at risk of renal function decline and arterial stiffening. Further studies are needed to confirm whether diagnosis of MetS using ABSI also predicts CVD morbidity and mortality.

## Supplementary information

Supplementary material

## Data Availability

The data that support the findings of this study are not publicly available because they contain information that could compromise the privacy of research participants. Further enquiries may be directed to the corresponding author.

## References

[1] Eckel RH, Grundy SM, Zimmet PZ (2005). The metabolic syndrome. The Lancet.

[2] Matsuzawa Y, Funahashi T, Nakamura T (2011). The concept of metabolic syndrome: contribution of visceral fat accumulation and its molecular mechanism. J Atheroscler Thromb.

[3] Isomaa B, Almgren P, Tuomi T, Forsén B, Lahti K, Nissén M (2001). Cardiovascular morbidity and mortality associated with the metabolic syndrome. Diabetes Care.

[4] Ninomiya T, Kubo M, Doi Y, Yonemoto K, Tanizaki Y, Rahman M (2007). Impact of metabolic syndrome on the development of cardiovascular disease in a general Japanese population: the Hisayama study. Stroke..

[5] Kurella M, Lo JC, Chertow GM (2005). Metabolic syndrome and the risk for chronic kidney disease among nondiabetic adults. J Am Soc Nephrol.

[6] Thomas G, Sehgal AR, Kashyap SR, Srinivas TR, Kirwan JP, Navaneethan SD (2011). Metabolic syndrome and kidney disease: a systematic review and meta-analysis. Clin J Am Soc Nephrol.

[7] Navaneethan SD, Schold JD, Kirwan JP, Arrigain S, Jolly SE, Poggio ED (2013). Metabolic syndrome, ESRD, and death in CKD. Clin J Am Soc Nephrol.

[8] Reaven GM (2006). The metabolic syndrome: is this diagnosis necessary?. Am J Clin Nutr.

[9] Sundström J, Vallhagen E, Risérus U, Byberg L, Zethelius B, Berne C (2006). Risk associated with the metabolic syndrome versus the sum of its individual components. Diabetes Care.

[10] Guembe MJ, Toledo E, Barba J, Martínez-Vila E, González-Diego P, Irimia P (2010). Association between metabolic syndrome or its components and asymptomatic cardiovascular disease in the RIVANA-study. Atherosclerosis.

[11] Zhang L, Zuo L, Wang F, Wang M, Wang S, Liu L (2007). Metabolic syndrome and chronic kidney disease in a Chinese population aged 40 years and older. Mayo Clin Proc.

[12] Ming J, Xu S, Yang C, Gao B, Wan Y, Xing Y (2014). China National Diabetes and Metabolic Disorders Study Group. Metabolic syndrome and chronic kidney disease in general Chinese adults: results from the 2007-08 China National Diabetes and Metabolic Disorders Study. Clin Chim Acta.

[13] Juonala M, Viikari JSA, Laitinen T, Marniemi J, Helenius H, Rönnemaa T (2004). Interrelations between brachial endothelial function and carotid intima-media thickness in young adults: the cardiovascular risk in young Finns study. Circulation.

[14] Nagayama D, Imamura H, Sato Y, Yamaguchi T, Ban N, Kawana H (2016). Inverse relationship of cardioankle vascular index with BMI in healthy Japanese subjects: a cross-sectional study. Vasc Health Risk Manag.

[15] Moore SC (2009). Waist versus weight: which matters more for mortality?. Am J Clin Nutr.

[16] Nagayama D, Watanabe Y, Yamaguchi T, Maruyama M, Saiki A, Shirai K (2020). New index of abdominal obesity, a body shape index, is BMI-independently associated with systemic arterial stiffness in real-world Japanese population. Int J Clin Pharmacol Ther.

[17] Topouchian J, Labat C, Gautier S, Bäck M, Achimastos A, Blacher J (2018). Effects of metabolic syndrome on arterial function in different age groups: the Advanced Approach to Arterial Stiffness study. J Hypertens.

[18] Gomez-Sanchez L, Garcia-Ortiz L, Patino-Alonso MC, Recio-Rodriguez JI, Fernando R, Marti R (2016). MARK Group. Association of metabolic syndrome and its components with arterial stiffness in Caucasian subjects of the MARK study: a cross-sectional trial. Cardiovasc Diabetol.

[19] Satoh N, Shimatsu A, Kato Y, Araki R, Koyama K, Okajima T (2008). Evaluation of the cardio-ankle vascular index, a new indicator of arterial stiffness independent of blood pressure, in obesity and metabolic syndrome. Hypertens Res.

[20] Christakoudi S, Tsilidis KK, Muller DC, Freisling H, Weiderpass E, Overvad K (2020). A Body Shape Index (ABSI) achieves better mortality risk stratification than alternative indices of abdominal obesity: results from a large European cohort. Sci Rep.

[21] Krakauer NY, Krakauer JC (2012). A new body shape index predicts mortality hazard independently of body mass index. PLoS One.

[22] Anoop S, Krakauer J, Krakauer N, Misra A (2020). A Body shape index significantly predicts MRI-defined abdominal adipose tissue depots in non-obese Asian Indians with type 2 diabetes mellitus. BMJ Open Diabetes Res Care.

[23] Liu J, Fan D, Wang X, Yin F (2020). Association of two novel adiposity indicators with visceral fat area in type 2 diabetic patients: Novel adiposity indexes for type 2 diabetes. Medicine.

[24] Bertoli S, Leone A, Krakauer NY, Bedogni G, Vanzulli A, Redaelli VI (2017). Association of Body Shape Index (ABSI) with cardiometabolic risk factors: A cross-sectional study of 6081 Caucasian adults. PLoS One.

[25] Ofstad AP, Sommer C, Birkeland KI, Bjørgaas MR, Gran JM, Gulseth HL (2019). Comparison of the associations between non-traditional and traditional indices of adiposity and cardiovascular mortality: an observational study of one million person-years of follow-up. Int J Obes..

[26] Teramoto T, Sasaki J, Ishibashi S, Birou S, Daida H, Dohi S (2013). Japan Atherosclerosis Society. Executive summary of the Japan Atherosclerosis Society (JAS) guidelines for the diagnosis and prevention of atherosclerotic cardiovascular diseases in Japan -2012 version. J Atheroscler Thromb.

[27] Matsuo S, Imai E, Horio M, Yasuda Y, Tomita K, Nitta K (2009). Revised equations for estimated GFR from serum creatinine in Japan. Am J Kidney Dis.

[28] Chapter 1: Definition and classification of CKD. Kidney Int Suppl (2011). 2013;3:19-62. 10.1038/kisup.2012.64. 10.1038/kisup.2012.64 PMC4089693 25018975

[29] Shirai K, Utino J, Otsuka K, Takata M (2006). A novel blood pressure independent arterial wall stiffness parameter; cardio-ankle vascular index (CAVI). J Atheroscler Thromb.

[30] Nakamura K, Tomaru T, Yamamura S, Miyashita Y, Shirai K, Noike H (2008). Cardio-ankle vascular index is a candidate predictor of coronary atherosclerosis. Circ J.

[31] Saiki A, Ohira M, Yamaguchi T, Nagayama D, Shimizu N, Shirai K (2020). New horizons of arterial stiffness developed using cardio-ankle vascular index (CAVI). J Atheroscler Thromb.

[32] Matsuzawa Y (2005). Metabolic syndrome - Definition and diagnostic criteria in Japan. J Atheroscler Thromb.

[33] Expert Panel on Detection, Evaluation, and Treatment of High Blood Cholesterol in Adults. Executive Summary of the Third Report of the National Cholesterol Education Program (NCEP) Expert Panel on Detection, Evaluation, and Treatment of High Blood Cholesterol in Adults (Adult Treatment Panal III). JAMA. 2001;285:2486-97. 10.1001/jama.285.19.2486 11368702

[34] Alberti KG, Zimmet P, Shaw J (2006). Metabolic syndrome–a new world-wide definition. A Consensus Statement from the International Diabetes Federation. Diabet Med.

[35] Grundy SM, Brewer HB, Cleeman JI, Smith SC, Lenfant C (2004). American Heart Association; National Heart, Lung, and Blood Institute. Definition of metabolic syndrome. Report of the National Heart, Lung and Blood Institute/American Heart Association conference on scientific issues related to definition. Circulation..

[36] Nagayama D, Watanabe Y, Saiki A, Shirai K, Tatsuno I (2018). Lipid parameters are independently associated with cardio-ankle vascular index (CAVI) in healthy Japanese subjects. J Atheroscler Thromb.

[37] Nagayama D, Watanabe Y, Saiki A, Shirai K, Tatsuno I (2019). Difference in positive relation between cardio-ankle vascular index (CAVI) and each of four blood pressure indices in real-world Japanese population. J Hum Hypertens.

[38] Sato Y, Nagayama D, Saiki A, Watanabe R, Watanabe Y, Imamura H (2016). Cardio-ankle vascular index is independently associated with future cardiovascular events in outpatients with metabolic disorders. J Atheroscler Thromb.

[39] Nakamura K, Iizuka T, Takahashi M, Shimizu K, Mikamo H, Nakagami T (2009). Association between cardio-ankle vascular index and serum cystatin C levels in patients with cardiovascular risk factor. J Atheroscler Thromb.

[40] Itano S, Yano Y, Nagasu H, Tomiyama H, Kanegae H, Makino H (2020). Association of arterial stiffness with kidney function among adults without chronic kidney disease. Am J Hypertens.

[41] Satirapoj B, Triwatana W, Supasyndh O (2020). Arterial stiffness predicts rapid decline in glomerular filtration rate among patients with high cardiovascular risks. J Atheroscler Thromb.

[42] Nagayama D, Endo K, Ohira M, Yamaguchi T, Ban N, Kawana H (2013). Effects of body weight reduction on cardio-ankle vascular index (CAVI). Obes Res Clin Pract.

[43] Examination Committee of Criteria for ‘Obesity Disease’ in Japan; Japan Society for the Study of Obesity. New criteria for ‘obesity disease’ in Japan. Circ J. 2002;66:987–92. 10.1253/circj.66.987. 10.1253/circj.66.987 12419927

[44] Onozaki A, Nagayama D, Azuma N, Sugai K, Shitara E, Sakai T (2021). Relation of maximum lifetime body mass index with age at hemodialysis initiation and vascular complications in Japan. Obes Facts.

[45] Xu T, Sheng Z, Yao L (2017). Obesity-related glomerulopathy: pathogenesis, pathologic, clinical characteristics and treatment. Front Med.

[46] Saiki A, Nagayama D, Ohhira M, Endoh K, Ohtsuka M, Koide N (2005). Effect of weight loss using formula diet on renal function for a short term in obese patients with diabetic nephropathy. Int J Obes.

[47] Harkins JM, Moustaid-Moussa N, Chung YJ, Penner KM, Pestka JJ, North CM (2004). Expression of interleukin-6 is greater in preadipocytes than in adipocytes of 3T3-L1 cells and C57BL/6J and ob/ob mice. J Nutr.

[48] Ferrara N, Davis-Smyth T (1997). The biology of vascular endothelial growth factor. Endocr Rev.

[49] Miricescu D, Balan DG, Tulin A, Stiru O, Vacaroiu IA, Mihai DA (2021). Impact of adipose tissue in chronic kidney disease development (Review). Exp Ther Med.

[50] Chughtai HL, Morgan TM, Rocco M, Stacey B, Brinkley TE, Ding J (2010). Renal sinus fat and poor blood pressure control in middle-aged and elderly individuals at risk for cardiovascular events. Hypertension..

[51] Sato Y, Fujimoto S, Konta T, Iseki K, Moriyama T, Yamagata K (2017). Body shape index: Sex-specific differences in predictive power for all-cause mortality in the Japanese population. PLoS One.

[52] Hemmelgarn BR, Manns BJ, Lloyd A, James MT, Klarenbach S, Quinn RR (2010). Alberta Kidney Disease Network. Relation between kidney function, proteinuria, and adverse outcomes. JAMA.

[53] Parker JL, Kirmiz S, Noyes SL, Davis AT, Babitz SK, Alter D (2020). Reliability of urinalysis for identification of proteinuria is reduced in the presence of other abnormalities including high specific gravity and hematuria. Urol Oncol.

