# Supplementary material for: Enhanced prediction of renal function decline by replacing waist circumference with “A Body Shape Index (ABSI)” in diagnosing metabolic syndrome: a retrospective cohort study in Japan
Source: Int J Obes (Lond). 2021 Nov 25;46(3):564–73. doi: 10.1038/s41366-021-01026-7 (PMC8872991; doi:10.1038/s41366-021-01026-7)
Supplement: Supplementary file 1 — Supplementary material [file 41366_2021_1026_MOESM1_ESM.docx]

**Table S1. Comparison of baseline clinical and biochemical characteristics in subjects with and those without renal function decline during 4-year study period.**

| Variables | Subjects without renal function decline  (N = 4,569) | Subjects with renal function decline  (N = 474) | *p* value |
| --- | --- | --- | --- |
| Male gender (%) | 41.8 | 50.4 | <0.001^*^ |
| Age (years) | 50 (42-58) | 73 (33-77) | <0.001 |
| Age ≥ 65 years (%) | 10.8 | 57.2 | <0.001^*^ |
| Current smoking (%) | 13.3 | 11.6 | 0.318* |
| Height (meter) | 1.62 (1.56-1.69) | 1.61 (1.52-1.67) | <0.001 |
| BMI (kg/m^2^) | 22.0 (20.1-24.1) | 21.8 (20.0-23.6) | 0.175 |
| BMI ≥ 25 kg/m^2^ (%) | 18.3 | 15.2 | 0.102^*^ |
| WC (meter) | 0.792 (0.731-0.852) | 0.797 (0.732-0.843) | 0.635 |
| ABSI | 0.0786 (0.0760-0.0815) | 0.0792 (0.0764-0.0834) | <0.001 |
| ABSI ≥ 0.080 (%) | 37.3 | 44.1 | 0.004^*^ |
| SBP (mmHg) | 116 (107-127) | 120 (109-133) | <0.001 |
| DBP (mmHg) | 73 (66-81) | 72 (66-79) | 0.007 |
| SBP≥130 and/or  DBP ≥ 85 mmHg (%) | 27.9 | 40.1 | <0.001^*^ |
| CAVI | 7.7 (7.2-8.3) | 8.7 (7.1-9.5) | <0.001 |
| CAVI ≥ 9.0 (%) | 9.3 | 43.2 | <0.001^*^ |
| FPG (mg/dL) | 87 (82-95) | 87 (82-93) | 0.238 |
| FPG ≥ 100 mg/dL (%) | 14.0 | 12.9 | 0.531 |
| FPG ≥ 110 mg/dL (%) | 6.4 | 4.6 | 0.136^*^ |
| LDL-C (mg/dL) | 127 (107-149) | 120 (99-141) | <0.001 |
| LDL-C ≥ 140 mg/dL (%) | 34.6 | 25.7 | <0.001^*^ |
| HDL-C (mg/dL) | 71 (59-84) | 64 (54-76) | <0.001 |
| TG (mg/dL) | 78 (57-113) | 80 (57-111) | 0.742 |
| TG > 150 and/or  HDL-C < 40 mg/dL (%) | 46.1 | 43.0 | 0.208^*^ |
| Creatinine (mg/dL) | 0.70 (0.60-0.83) | 0.74 (0.62-0.86) | <0.001 |
| eGFR (mL/min/1.73m^2^) | 77.5 (69.2-86.8) | 75.4 (64.5-86.8) | <0.001 |
| Japanese WC-MetS (%) | 5.4 | 4.9 | 0.669^*^ |
| Japanese ABSI-MetS (%) | 7.4 | 16.5 | <0.001^*^ |
| IDF WC-MetS (%) | 20.6 | 25.3 | 0.018^*^ |
| IDF ABSI-MetS (%) | 9.4 | 15.4 | <0.001^*^ |
| NCEP ATPIII WC-MetS (%) | 4.4 | 4.0 | 0.813^*^ |
| NCEP ATPIII ABSI-MetS (%) | 11.6 | 16.9 | 0.001^*^ |

Data are presented as median interquartile range. Mann-Whitney U test and *Fisher’s exact test were used to compare subjects with or without developing renal function decline defined as eGFR < 60 mL/min/1.73m^2^ during the 4-year study period. BMI, body mass index; WC, weight circumference; ABSI, a body shape index; SBP, systolic blood pressure; DBP, diastolic blood pressure; CAVI, cardio-ankle vascular index; FPG, fasting plasma glucose; LDL-C, low-density lipoprotein-cholesterol; HDL-C, high-density lipoprotein-cholesterol; TG, triglyceride; eGFR, estimated glomerular filtration rate; WC-MetS, conventional metabolic syndrome (MetS) diagnosed using waist circumference (WC); ABSI-MetS, MetS diagnosed using a body shape index (ABSI) instead of WC; Japanese, criteria developed by the Japanese Committee for the Diagnostic Criteria of MetS; IDF, International Diabetes Federation; NCEP-ATPIII; National Cholesterol Education Program Adult Treatment Panel III.

**Table S2. Survival rate (95% confidence intervals) for renal function decline at each observation period.**

|  |  | Year of follow-up | | | |
| --- | --- | --- | --- | --- | --- |
|  |  | 1 | 2 | 3 | 4 |
| (A) Japanese WC-MetS | Yes | 0.978 (0.951-0.990) | 0.945 (0.910-0.966) | 0.915 (0.875-0.943) | 0.915 (0.875-0.943) |
|  | No | 0.973 (0.968-0.977) | 0.939 (0.932-0.945) | 0.917 (0.908-0.924) | 0.905 (0.897-0.913) |
| (B) IDF WC-MetS | Yes | 0.968 (0.955-0.977) | 0.922 (0.904-0.936) | 0.887 (0.866-0.904) | 0.887 (0.866-0.904) |
|  | No | 0.974 (0.969-0.979) | 0.944 (0.936-0.951) | 0.924 (0.916-0.932) | 0.911 (0.902-0.920) |
| (C) NCEP-ATPⅢ WC-MetS | Yes | 0.964 (0.936-0.980) | 0.922 (0.886-0.947) | 0.877 (0.834-0.909) | 0.877 (0.834-0.909) |
|  | No | 0.973 (0.968-0.978) | 0.940 (0.933-0.947) | 0.919 (0.911-0.927) | 0.908 (0.899-0.916) |
| (D) Japanese ABSI-MetS | Yes | 0.959 (0.935-0.974) | 0.873 (0.837-0.901) | 0.812 (0.772-0.847) | 0.812 (0.772-0.847) |
|  | No | 0.974 (0.969-0.978) | 0.945 (0.938-0.952) | 0.926 (0.918-0.933) | 0.914 (0.906-0.922) |
| (E) IDF ABSI-MetS | Yes | 0.966 (0.946-0.979) | 0.912 (0.884-0.934) | 0.855 (0.821-0.883) | 0.855 (0.821-0.883) |
|  | No | 0.974 (0.968-0.978) | 0.942 (0.935-0.949) | 0.923 (0.915-0.931) | 0.912 (0.903-0.920) |
| (F) NCEP-ATPⅢ ABSI-MetS | Yes | 0.966 (0.948-0.977) | 0.916 (0.891-0.936) | 0.869 (0.839-0.893) | 0.869 (0.839-0.893) |
|  | No | 0.974 (0.969-0.978) | 0.942 (0.935-0.949) | 0.923 (0.915-0.931) | 0.911 (0.902-0.919) |

Kaplan-Meier survival analyses for the occurrence of renal function decline when MetS was diagnosed by various criteria.

WC-MetS, conventional metabolic syndrome (MetS) diagnosed using waist circumference (WC); ABSI-MetS, MetS diagnosed using a body shape index (ABSI) instead of WC; Japanese, criteria developed by the Japanese Committee for the Diagnostic Criteria of MetS; IDF, International Diabetes Federation; NCEP-ATPIII; National Cholesterol Education Program Adult Treatment Panel III.
